# Validation of the Edinburgh postpartum depression scale in a population of puerperal women in Mexico

**DOI:** 10.1186/1745-0179-2-33

**Published:** 2006-11-29

**Authors:** Cosme Alvarado-Esquivel, Antonio Sifuentes-Alvarez, Carlos Salas-Martinez, Sergio Martínez-García

**Affiliations:** 1 Facultad de Medicina, Universidad Juárez del Estado de Durango. Durango, Dgo. México; 2 Hospital General. Secretaría de Salud. Durango, Dgo. México

## Abstract

**Background:**

The Edinburgh postnatal depression scale (EPDS) has been validated and used successfully in detecting postnatal depression in several language versions in a number of countries. However, there is not any Mexican version of the EPDS that had been validated. Therefore, we sought to validate a Spanish translated Mexican version of the EPDS in a population of puerperal Mexican women.

**Methods:**

One hundred puerperal women within their three month postpartum period attending routine postnatal consultations in a public hospital in Durango City, Mexico participated in the study. The participants were divided into two groups: one group included 49 women with less than 4 weeks of postpartum, and the other group included 51 women within 4 to 13 weeks of postpartum. All participants submitted a Spanish translated Mexican version of the EPDS and were interviewed by a psychiatrist to assess major and minor depression by using DSM-IV criteria.

**Results:**

Out of the 49 women with less than 4 weeks of postpartum, 4 were found as suffering from major depression and none from minor depression by using the DSM-IV criteria. In this group of women we found that the best EPDS score for screening depression was 11/12. This threshold showed a sensitivity of 75% (95% CI: 63.8–86.2), a specificity of 93% (95% CI: 84.6–100), a positive predictive value of 50%, a negative predictive value of 97.6%, and an area under the curve of 0.84. While in the 51 women within 4 to 13 weeks of postpartum, 7 were found as suffering from major depression and 1 from minor depression by using the DSM-IV criteria. In this group we found that the best EPDS score for screening depression was 7/8. This threshold showed a sensitivity of 75% (95% CI: 66.1–83.9), a specificity of 84% (95% CI: 76.1–91.9), a positive predictive value of 46.2%, a negative predictive value of 94.7% and an area under the curve of 0.80.

**Conclusion:**

The Mexican version of the EPDS can be considered for screening depression in puerperal Mexican women whenever cut-off scores of 11/12 and 7/8 in women with less than 4 weeks and within 4 to 13 weeks of postpartum are used, respectively.

## Background

Postnatal depression is the most common complication of childbearing [1] affecting from 10 to 20% of women [1-3]. The Edinburgh postnatal depression scale (EPDS) is a validated instrument developed specifically to identify women experiencing postnatal depression [4,5]. The EPDS has been translated from the original English version [4] to a number of languages and has been validated and used successfully in detecting postnatal depression in a number of countries [6-10]. However, validations studies have shown an important variability in sensitivity, specificity and optimal cut-off scores of the instrument in populations of several countries [6-10]. In addition, the optimal cut-off score varies in prenatal, early postpartum and late postpartum [6-11]. Therefore, validation of the EPDS in particular populations is highly recommended before the instrument can be used for screening depression. In Mexico, there is not any Mexican version of the EPDS that had been validated. Therefore, we sought to validate a Spanish translated Mexican version of the EPDS in a population of puerperal Mexican women. The validation of this Mexican version of the EPDS has an epidemiological impact since it is the first logical step to be performed just before the instrument can be used in screening studies of postnatal depression in Mexico. The use of this validated Mexican version may contribute to determine the magnitude of postnatal depression in Mexico.

## Methods

### Study population

One hundred puerperal women attending routine postnatal consultations in a public hospital in Durango City, Mexico participated in the study. Women belonged to a low socioeconomic status, the majority of them resided in Durango City and the rest in neighboring towns around Durango City. Women were invited to participate when they attended their postnatal appointments as a regular clinical practice for check up after childbirth. Inclusion criteria for enrollment in the study were puerperal women within their 3 month postpartum period and acceptance to participate in the study. Participants were enrolled consecutively. The participants were divided into two groups: one group included 49 women with less than 4 weeks of postpartum, and the other group included 51 women within 4 to 13 weeks of postpartum. The women with less than 4 weeks of postpartum had a mean age of 23.9 years old (range 15–32 years), and were evaluated once at a mean of 1.8 weeks after childbirth. Out of the 49 women of this group, 41 had finished their last pregnancy by cesarean section and 8 by vaginal delivery. Participating women of this group had had a mean number of pregnancies of 2.1 (range 1 to 5). The women within 4 to 13 weeks of postpartum had a mean age of 24.7 years old (range 17–35 years), and were evaluated once at a mean of 5.5 weeks after childbirth. Out of the 51 women of this group, 15 had finished their last pregnancy by cesarean section and 36 by vaginal delivery. Participating women of this group had had a mean number of pregnancies of 2.2 (range 1 to 7).

### Evaluation of the Edinburgh postnatal depression scale

The Mexican version of the EPDS [see Additional file 1] was constructed from the original English version [4] and a Spanish version [9] of the instrument. Since the Spanish version of the EPDS contains a number of words not currently used in Mexico, we have changed those words for a more colloquial ones used for the general population of Mexico. Nevertheless, the meaning of the words and the general structure of the newly created Mexican version of the instrument are in close agreement with those of the original English version. Two bilingual professors performed reverse translations of the Mexican version of the EPDS into English and accuracy was confirmed. As a gold standard for diagnosing depression we used the DSM-IV criteria for major and minor depression [12]. We used these criteria because they have been successfully used by other researchers in similar studies [6,10,13,14]. All participants were asked to complete the self administered Mexican version of the EPDS and then were interviewed by a psychiatrist to assess major and minor depression by using the DSM-IV criteria. Both EPDS and psychiatric interview were performed during the same day to each woman. Psychiatric interview was performed by one psychiatrist (CSM). EPDS scores were not provided to the psychiatrist, and analysis of the data was performed by persons (CAE, SMG) other than the psychiatrist (CSM) who performed the interview and the gynecologist (ASA) who applied the EPDS.

### Statistical analysis

Statistical analysis was performed with the aid of the software Microsoft Excel. Sensitivity, specificity, and positive and negative predictive values of the evaluated instrument were obtained. The best cut-off scores of the Mexican version of the EPDS for screening depression in the puerperal women were obtained by drawing receiver operating characteristic curves.

## Results

### Women with less than 4 weeks of postpartum

**Figure 1:**
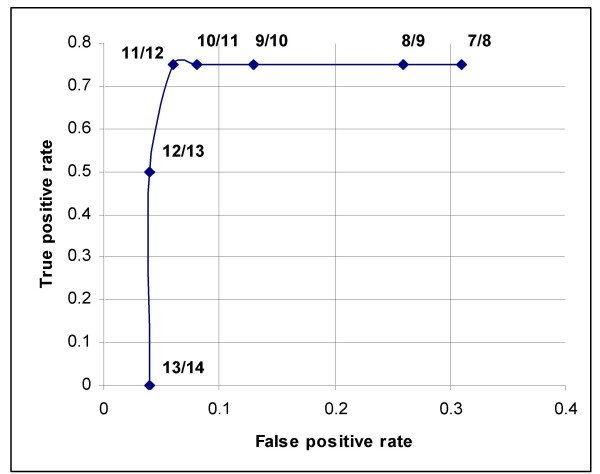
A receiver operating characteristic curve that shows different cut-off points of the EPDS in women with less than 4 weeks of postpartum. Good performance of the Mexican version of the EPDS in these women was found at 11/12 cut-off point.

Out of the 49 puerperal women with less than 4 weeks of postpartum, 4 had major depression and none had minor depression according to the DSM-IV criteria. Results of sensitivity and specificity for different EPDS scores found in the 49 women of this group are shown in Table 1. As seen in Figure 1, the receiver operating characteristic curve showed that the best sensitivity and specificity of the Mexican version of the EPDS in this group of women with less than 4 weeks of postpartum was found at 11/12 score. At this threshold, we found a sensitivity of 75% (95% CI: 63.8–86.2), and a specificity of 93% (95% CI: 84.6–100). The area under the curve was 0.84. Increasing the threshold to 12/13 the sensitivity was reduced to 50% but the specificity increased to 96%. While lowering the threshold to 10/11 the sensitivity remained at 75% but the specificity was reduced to 91%. Out of the 4 women with depression by the DSM-IV criteria, 3 were positive and 1 negative in the EPDS. While out of the 45 women without depression by the DSM-IV criteria, 42 were negative and 3 positive in the EPDS. Thus a positive predictive value of 50% and a negative predictive value of 97.6% for the EPDS was obtained. Depressed women were treated either with Fluoxetine 20 mg/day or psychotherapy.

**Table 1:** Sensitivity and specificity of the Mexican version of EPDS at different thresholds as compared with DSM-IV results in women with less than 4 weeks of postpartum.

EPDS Score	Sensitivity %	Specificity %
0–1	100.00	13.00
1–2	100.00	24.00
2–3	100.00	27.00
3–4	75.00	36.00
4–5	75.00	44.00
5–6	75.00	49.00
6–7	75.00	56.00
7–8	75.00	69.00
8–9	75.00	73.00
9–10	75.00	87.00
10–11	75.00	91.00
**11–12**	75.00	93.00
12–13	50.00	96.00
13–14	0.00	96.00
14–15	0.00	98.00
15–16	0.00	98.00
16–17	0.00	98.00
17–18	0.00	100.00
18–19	0.00	100.00
19–20	0.00	100.00

### Women within 4 to 13 weeks of postpartum

**Figure 2:**
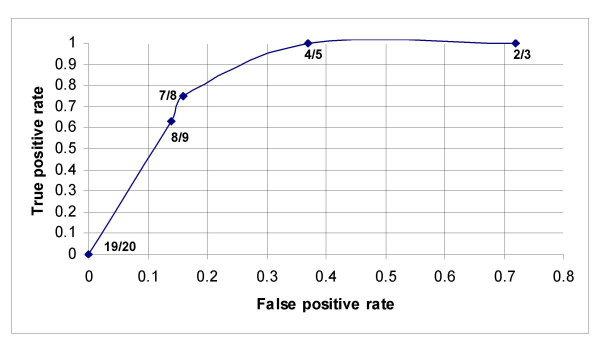
A receiver operating characteristic curve that shows different cut-off points of the EPDS in women within 4 to 13 weeks of postpartum. Good performance of the Mexican version of the EPDS in these women was found at 7/8 cut-off point.

Out of the 51 puerperal women within 4 to 13 weeks of postpartum, 7 had major depression and 1 had minor depression according to the DSM-IV criteria. Results of sensitivity and specificity for different EPDS scores found in the 51 women of this group are shown in Table 2. As seen in Figure 2, the receiver operating characteristic curve showed that the best sensitivity and specificity of the Mexican version of the EPDS in this group was found at 7/8 score. At this threshold, we found a sensitivity of 75% (95% CI: 66.1–83.9), and a specificity of 84% (76.1–91.9). The area under the curve was 0.80. Increasing the threshold to 8/9 the sensitivity was reduced to 63% but the specificity increased slightly to 86%. While lowering the threshold to 6/7 the sensitivity remained at 75% but the specificity was reduced to 72%. Out of the 8 women with depression by the DSM-IV criteria, 6 were positive and 2 negative in the EPDS. While out of the 43 women without depression by the DSM-IV criteria, 36 were negative and 7 positive in the EPDS. Thus a positive predictive value of 46.2% and a negative predictive value of 94.7% for the EPDS was obtained. Depressed women were treated with Sertraline 50 mg/day, or Fluoxetine 20 mg/day.

**Table 2:** Sensitivity and specificity of the Mexican version of EPDS at different thresholds as compared with DSM-IV results in women within 4 to 13 weeks of postpartum.

EPDS score	Sensitivity %	Specificity %
0–1	100.00	14.00
1–2	100.00	21.00
2–3	100.00	28.00
3–4	100.00	40.00
4–5	100.00	63.00
5–6	100.00	70.00
6–7	75.00	72.00
**7–8**	75.00	84.00
8–9	63.00	86.00
9–10	63.00	86.00
10–11	63.00	95.00
11–12	38.00	95.00
12–13	38.00	98.00
13–14	38.00	98.00
14–15	38.00	100.00
15–16	38.00	100.00
16–17	38.00	100.00
17–18	25.00	100.00
18–19	13.00	100.00
19–20	0.00	100.00

## Discussion

Validation of the EPDS is an important procedure that provides reliability of the instrument when a version other than the original one is used. We found that the Mexican version of the EPDS can be successfully used to screen depression in a Mexican population of puerperal women during their three month postnatal period. However, this instrument performed good only when thresholds of 11/12 and 7/8 in women with less than 4 weeks and women within 4 to 13 weeks of postpartum were used, respectively. The 7/8 threshold is rather low as compared with those used in other EPDS versions. For instance, the 7/8 threshold found in our study differs from that described in the original English version of the EPDS [4]. The original version successfully detected depressed women when a threshold score of 12/13 was used [4]. Although the optimal cut-off score of 7/8 obtained in the Mexican version of the EPDS is lower than that of 12/13 found in the original version, a comparable sensitivity and specificity among both studies is observed. The optimal threshold of 12/13 reported in the original version of the EPDS has a sensitivity of 86% and a specificity of 78% [4] while we found that our 7/8 score has a sensitivity of 75% and a specificity of 84%. Like ours other validation studies have found optimal EPDS scores also different from that described in the original version of the EPDS. The difference in the EPDS scores among the studies does not only depend on differences in language or culture of the populations but also depends on differences in the time of assessment in relation to childbirth (antenatal, early postpartum or late postpartum). For instance, a validation study performed in France with women attending antenatal consultations for pregnancy complication found that 11.5 was the optimal cut-off score [6]. In Norway, a study in women at 6–12 weeks postpartum found that a cut-off of 11 has a sensitivity of 96% and a specificity of 78% [8]. In south Africa, a threshold of 11/12 on the EPDS was the best to screen depression in women attending a postnatal clinic [13]. In contrast, in a study performed in women at their third to fifth day postpartum, researchers found an EPDS sensitivity of 82% with a positivity threshold of 9.5/30 [11]. Similarly, when the EPDS score found in the Mexican version was compared with those described in other Spanish versions of the instrument, we found that the EPDS score found in our study was lower than those reported in Spain [9] and Peru [15]. In Spain the best threshold for EPDS in women attending a routine postnatal check-up at 6 weeks postpartum was found at 10/11, while in Peru the best threshold was found at 13.5 in women within the first year of postpartum. The low threshold obtained in our study is the lowest reported. There is only one validation study performed in India that reported a comparable threshold that the one found in our study. In India, researchers found an optimal threshold of 8/9 in women in the postpartum period [16]. Results of validations studies [6-9,14] indicate that optimal threshold of EPDS for screening depression is quite variable depending on the population studied, and this variability occurs even when the instrument is used in the same language. Thus our results and those obtained in other studies indicate the need to perform evaluations of the EPDS in each country before the instrument can be used for screening depression. Only with this practice, screening studies in particular populations may reach reliable results.

## Conclusion

The Mexican version of the EPDS performs good for screening depression in Mexican puerperal women. Best EPDS cut-off scores varied depending on the weeks after childbirth. Recommended EPDS cut-off scores in Mexican women are 11/12 for women with less than 4 weeks of postpartum, and 7/8 for women within 4 to 13 weeks of postpartum.

## Competing interests

The author(s) declare that they have no competing interests.

## Authors' contributions

CAE conceived and designed the study protocol, participated in the coordination and management of the study, performed the data analysis and wrote the manuscript. ASA applied the questionnaires and obtained the general data of the participants. CSM performed the clinical evaluation of the participants. SMG performed the data analysis. All authors read and approved the final manuscript.

## Supplementary Material

Additional file 1 Mexican version of the EPDS. The data provided is the Spanish translated Mexican version of the EPDS that consists of 10 questions and 4 response categories. Click here for file
