# Supplementary material for: Validation of the Edinburgh postpartum depression scale in a population of puerperal women in Mexico
Source: Clin Pract Epidemiol Ment Health. 2006 Nov 29;2:33. doi: 10.1186/1745-0179-2-33 (PMC1693550; doi:10.1186/1745-0179-2-33)
Supplement: Additional file 1 — Mexican version of the EPDS. The data provided is the Spanish translated Mexican version of the EPDS that consists of 10 questions and 4 response categories. [file 1745-0179-2-33-S1.doc]

**Additional file 1. Mexican version of the EPDS.**

Como usted hace poco tuvo un bebe, nos gustaría saber como se ha estado sintiendo. Por favor **subraye** la respuesta que más se acerca a como se ha sentido **en los últimos 7 días**.

1. He podido reír y ver el lado bueno de las cosas:

Tanto como siempre 0

No tanto ahora 1

Mucho menos 2

No, no he podido. 3

2. He mirado el futuro con placer:

Tanto como siempre 0

Algo menos que antes 1

Definitivamente menos que antes 2

No, nada 3

3. Me he culpado innecesariamente cuando las cosas marchaban mal:

Sí, la mayoría de las veces 3

Si, algunas veces 2

No muy a frecuentemente 1

No, nunca 0

4. He estado ansiosa y preocupada sin motivo:

No, nada 0

Rara vez 1

Sí, a veces 2

Sí, muy frecuentemente 3

5. He sentido miedo o pánico sin motivo alguno:

Sí, muy frecuentemente 3

Sí, a veces 2

No, no mucho 1

No, nada 0

6. Las cosas me han estado agobiando:

Sí, casi siempre 3

Sí, a veces 2

No, casi nunca 1

No, nada 0

7. Me he sentido tan infeliz, que he tenido dificultad para dormir:

Sí, casi siempre 3

Sí, a veces 2

No muy frecuentemente 1

No, nada 0

8. Me he sentido triste y desgraciada:

Sí, casi siempre 3

Sí, muy frecuentemente 2

No muy frecuentemente 1

No, nada 0

9. He estado tan infeliz que he estado llorando:

Sí, casi siempre 3

Sí, muy frecuentemente 2

Sólo ocasionalmente 1

No, nunca 0

10. He pensado en hacerme daño a mi misma:

Sí, muy frecuentemente 3

A veces 2

Rara vez 1

Nunca 0
